# Case Report: Coexistence of Anti-AMPA Receptor Encephalitis and Positive Biomarkers of Alzheimer's Disease

**DOI:** 10.3389/fneur.2021.673347

**Published:** 2021-07-02

**Authors:** Yu Song, Shanshan Chen, Ju Gao, Jie Lu, Wenwen Xu, Xingjian Lin, Jiu Chen

**Affiliations:** ^1^Department of Neurology, The Affiliated Brain Hospital of Nanjing Medical University, Nanjing, China; ^2^Department of Geriatric Psychiatry, The Affiliated Brain Hospital of Nanjing Medical University, Nanjing, China; ^3^Institute of Neuropsychiatry, The Affiliated Brain Hospital of Nanjing Medical University, Nanjing, China; ^4^Institute of Brain Functional Imaging, Nanjing Medical University, Nanjing, China

**Keywords:** autoimmune encephalitis, anti-α-amino-3-hydroxy-5-methyl-4-lsoxazolepropionic acid receptor, Alzheimer's disease, cognitive impairment, cerebrospinal fluid biomarkers

## Abstract

Anti–α-amino-3-hydroxy-5-methyl-4-isoxazolepropionic acid (AMPA) receptor encephalitis is a rare autoimmune disease that is characterized by acute cognitive impairment, mental symptoms, and seizures. The high comorbidity rate between anti–AMPA receptor (AMPAR) encephalitis and other somatic diseases, such as malignancy, has revealed the possibility of potential copathogenesis. However, there have not yet been reports about anti-AMPAR encephalitis with concomitant cerebrospinal fluid (CSF) biomarkers consistent with Alzheimer disease (AD). Herein, we present the case of an elderly male patient with autoimmune encephalitis (AE) presenting with anti–AMPA1-R and anti–AMPA2-R antibodies, as well as CSF biomarkers of AD. The patient was hospitalized with acute memory decline for 1 week. Anti–AMPA1-R and anti–AMPA2-R antibodies were positively detected in CSF, and the anti–AMPA2-R antibody was also present in the serum. Additionally, the biomarkers of AD were concurrently present in CSF (Aβ_1−42_ = 245.70 pg/mL, t-Tau = 894.48 pg/mL, p-Tau = 78.66 pg/mL). After administering a combined treatment of intravenous immunoglobulin and glucocorticoids, the patient recovered significantly, and his cognitive function achieved a sustained remission during 2 months' follow-up. This case raises the awareness of a possible interaction between AE and changes of CSF biomarkers. We speculated that the existence of AMPAR antibodies can induce changes of CSF, and other pathological alterations. This present report highlights that a potential relationship exists among AE and provides a warning when making the diagnosis of AD.

## Background

Autoimmune encephalitis (AE) is defined as a group of important neurological inflammatory diseases with specific autoantibodies. The incidence of AE has increased to 1.2/100,000 person-years (2006–2015) compared to 0.4/100,000 person-years (1995–2005) (1). The rapid development of a spectrum of specific autoantibody-associated neurological disorders has deepened our understanding in the last 30 years. As one of the specific antibodies targeting neuronal surface antigens, which are more likely to be pathogenic, anti–α-amino-3-hydroxy-5-methyl-4-isoxazolepropionic acid receptor (AMPAR) antibody has rarely been seen in clinical practice. To date, ~60 related cases have been reported (2). Anti-AMPAR encephalitis is characterized by diverse clinical manifestations, and ~60% of patients might be associated with malignancy (3). Coexisting antibodies, such as collapsin response mediator protein 5 antibody, have also been identified (4). Nevertheless, to the best of our knowledge, no case has ever been reported, comprising two positive subtypes of anti-AMPAR antibodies and typical changes of biomarkers of Alzheimer disease (AD).

Herein, we report the case of a 79-year-old man diagnosed with anti-AMPAR encephalitis with the coexistence of antibodies targeting AMPA1 receptor (AMPA1-R) and AMPA2 receptor (AMPAR2-R) and positive cerebrospinal fluid (CSF) biomarkers of AD, manifested as rapidly progressive dementia. We aim to explore the underlying pathological mechanisms of AE and the CSF biomarkers of AD.

## Case Presentation

A 79-year-old man was admitted to the Nanjing Brain Hospital with rapid memory decline for 1 week, which aggravated in the past 2 days on October 16, 2020. The patient especially had deficits in recent memory, such as forgetting what he had just done or said and occasionally did not recognize the family members. His symptoms were repetitive, with remissions and exacerbations. Two days before admission, the cognitive function had further declined, mainly manifested as failure to recognize family members and inability to take care of himself. Thus, he was admitted to the hospital, brought by his son. Five days after admission, the patient developed mental symptoms, the hallmark of which was visual hallucinations. The family members reported that the patient saw people or things that did not exist and mistook the hospital for the street. During the disease course, the patient did not undergo epileptic seizures.

The patient had a history of diabetes for 2 years, which was treated by the oral administration of metformin hydrochloride (Diaformin) tablets (1.0 g, twice a day) and acarbose tablets (50 mg, thrice a day). He also had hypertension for several years and was treated by the oral administration of telmisartan tablets (40 mg, once a day), with no history of anxiety, depression, or epilepsy. However, the blood sugar levels and blood pressure were not monitored regularly. The patient lived with his wife all year round. A week before admission, his children noticed that his memory was significantly worse than before when they visited him. His wife reported that in the last 2 years, he sometimes forgot to buy things, but their life was not affected. There were no cases with similar symptoms or related family history of autoimmune diseases and dementia.

**Figure 1:**
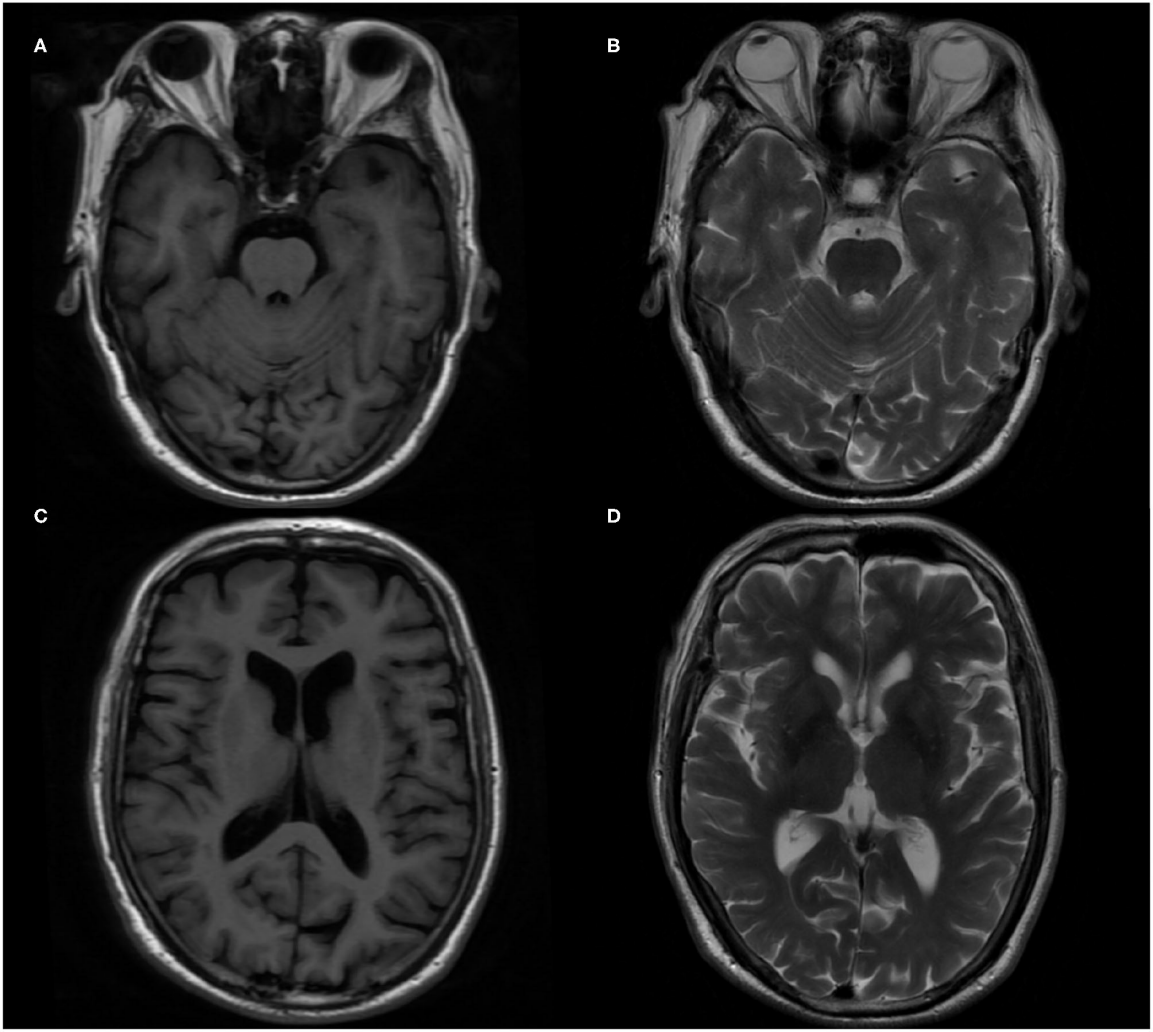
Brain magnetic resonance imaging (MRI) in the patient. **(A,C)** T1-weighted imaging axial image. **(B,D)** T2-weighted imaging axial image.

In terms of tests, cranial magnetic resonance imaging (MRI) revealed ischemic changes in centrum semiovale and corona radiate, with no other abnormalities, particularly no signs of limbic encephalitis (i.e., hippocampal sclerosis) (Figure 1). Electroencephalography (EEG) showed an extensive moderate abnormality, with no epileptiform discharge. Chest computed tomography (CT) showed a nodular hyperdense shadow with a diameter of ~4.6 mm within the upper lobe of the right lung. The patient underwent neuropsychological evaluations the day after admission, in which he scored 2/30 on the Mini-Mental State Examination (MMSE), 1/30 on the Montreal Cognitive Assessment (MoCA), and 4.5/32.5 on the Hasegawa Dementia Scale. The patient also underwent laboratory tests. His hemoglobin A_1c_ level was 6.30%. Furthermore, his CSF exhibited increased protein levels (0.52 g/L, normal range = 0.20–0.40 g/L) and immunoglobulin G (46.1 mg/L, normal range = 0–34 mg/L). Routine blood and other CSF analyses (cell counts, glucose, chlorine level, and pathology) were normal. CSF polymerase chain reaction for herpes simplex viruses 1 and 2 was unremarkable. Anti–AMPA1-R and anti–AMPA2-R antibodies were detected in the CSF (titer: 1:3.2 and 1:320), and anti–AMPA2-R antibody was also present in the serum (titer: 1:100). Simultaneously, AD biomarkers were present in the CSF: Aβ_1−42_ = 245.70 pg/mL, t-Tau = 894.48 pg/mL, and p-Tau = 78.66 pg/mL (Table 1). Besides, urinary AD-associated neuronal thread protein, tumor markers, paraneoplastic neuronal antibodies, and other common antineuronal and antineuropil antibodies did not exhibit significant changes. Other laboratory tests, such as thyroid hormone combination, the spectrum of antinuclear antibodies, and anticardiolipin antibodies, were normal. During the hospitalization, the patient's blood pressure and blood glucose were well-controlled, with an average blood pressure of 136/92 mm Hg and fasting blood glucose of ~5.89 mmol/L. All the serum and CSF antibodies were measured using a fixed cell-based assay, and the quantitative determination of CSF biomarkers was carried out using an enzyme-linked immunosorbent assay. All the tests were carried out twice.

**Table 1:** Test results of CSF biomarkers of AD.

**Test method**	**Results**		**Reference interval**	
ELISA	Aβ_1−42_	↓245.70 pg/mL	<550 pg/mL	Aggregated Aβ
			551–650 pg/mL	Suspicious
			≥651 pg/mL	Normal
ELISA	Aβ_1−40_	8,046.11 pg/mL	≥7,000 pg/mL	Normal
			<7,000 pg/mL	Aggregated Aβ
ELISA	Aβ_1−42_/Aβ_1−40_	↓0.031	≤ 0.05	Positive
			>0.05	Negative
ELISA	t-Tau	↑894.48 pg/mL	≤ 399 pg/mL	Normal
			>399 pg/mL	Neurodegeneration or neuronal injury
ELISA	p-Tau	↑78.66 pg/mL	≤ 50 pg/mL	Normal
			>50 pg/mL	Neurofibrillary tangles

*CSF, cerebrospinal fluid; AD, Alzheimer disease; ELISA, enzyme-linked immunosorbent assay; Aβ, amyloid β-protein; t-Tau, total-Tau protein; p-Tau, phosphorylated-Tau protein. The method of the ELISA determinations can be seen in the Supplementary Material*.

*↓: decreased; ↑: increased*.

Because of the history of diabetes and hypertension, cerebrovascular diseases had to be considered. However, the results of brain MRI and CT excluded any possibility. Although a nodular high-density shadow was seen in the right lung, the patient had no cough, coughing sputum, hemoptysis, and no enlargement of superficial lymph nodes throughout the body. Tumor markers were all normal. After consultation with a respiratory specialist, the tumor possibility was considered unlikely, and regular follow-up was recommended.

**Figure 2:**
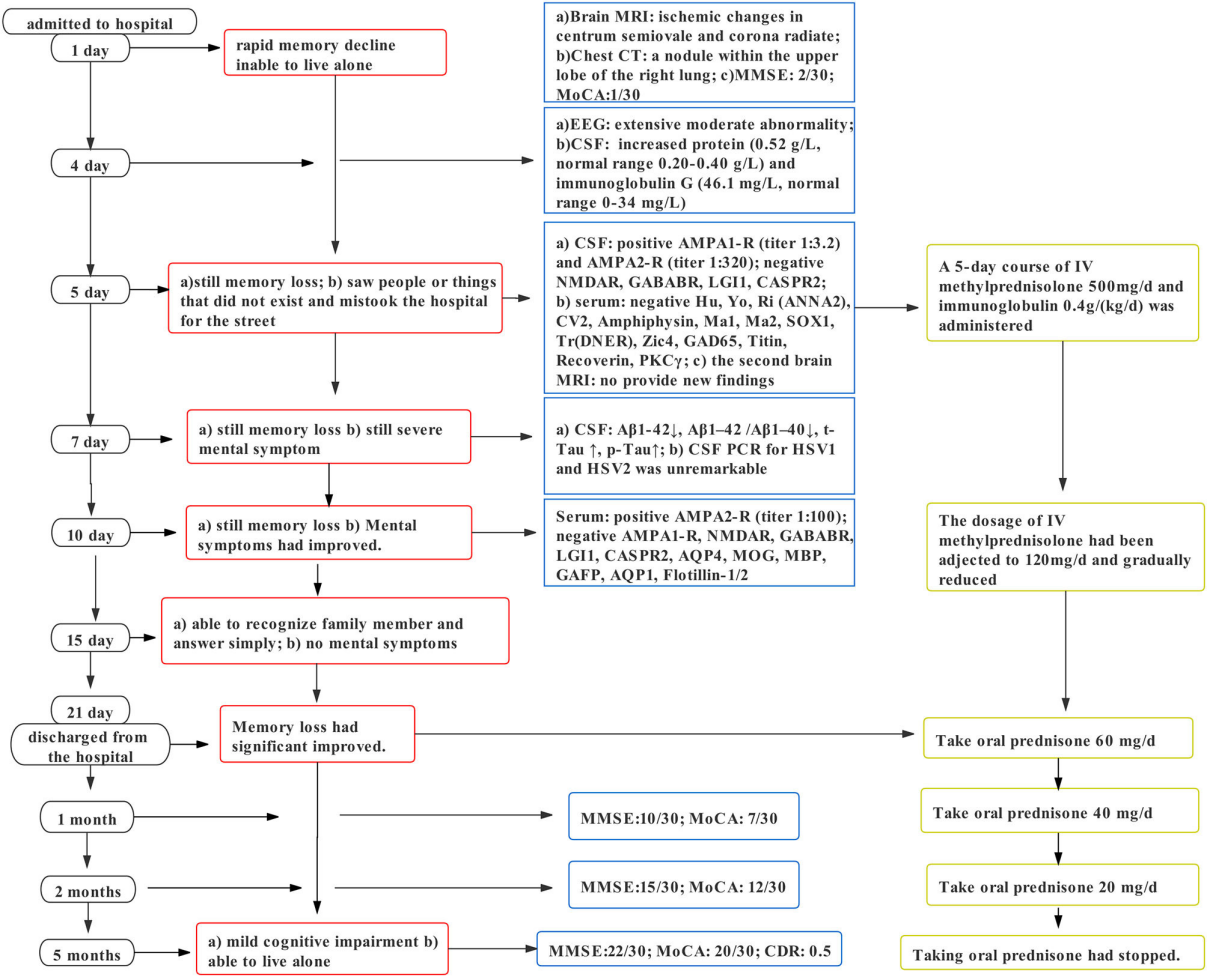
Clinical course of the case.

The results of autoimmune antibodies were available 5 days after admission; the patient was diagnosed with anti-AMPAR encephalitis. The sign of the tumor was ruled out, and immunotherapy was initiated with intravenous methylprednisolone (500 mg/d) and immunoglobulin 0.4 g/kg per day for 5 days, and then methylprednisolone dose was gradually reduced. Two days later, an analysis of the CSF biomarkers showed amyloid and Tau abnormalities. Uncertain whether AE caused changes in the CSF biomarkers or whether there existed concomitant chronic cognitive decline, we planned to treat the patient with immunotherapy first and then determine the need for other drugs such as cholinesterase inhibitors based on the patient's subsequent recovery. Ten days after treatment, he recovered significantly but still suffered from mild memory impairment. Finally, he was discharged with 60 mg/d prednisone, Caltrate, and potassium chloride sustained-release tablets to prevent the side effects of the glucocorticoid. Thirty days later, the patient's cognitive function improved as he scored 10/30 on MMSE and 7/30 on MoCA. During the 2-month follow-up, his prednisolone acetate tablets had been adjusted to 20 mg/d with cognitive scale assessment: MMSE: 15/30 and MoCA: 12/30. Five months after discharge, the patient stopped taking oral prednisolone and the related medications used to prevent the side effects of the glucocorticoid and scored 22/30 on MMSE, 20/30 on MoCA, and 0.5 on the clinical dementia rating. Confirmed by his son, the patient could live alone and forgot things occasionally. We recommended that the patient should still perform appropriate cognitive function exercises daily and be followed regularly, with additional pharmacological interventions if necessary. Figure 2 summarizes the clinical course. Details about the laboratory evaluations are presented in the Supplementary Material.

## Discussion and Conclusion

Since the characterization of anti-NMDAR encephalitis in 2007, more types of AE have been explored. However, anti-AMPAR encephalitis is a relatively rare type. Because of a lack of relevant case reports and large-scale studies, the clinical manifestations and treatments have not been consistent (5). Although the core symptoms of anti-AMPAR encephalitis include cognitive impairment, mental symptoms, and seizures, some patients might present motor–sensory disturbances, ataxic gait, or dizziness (6). Currently, immunocytochemistry on HEK 293 cells transfected with GluA1 and / or GluA2 subunits is mainly used to diagnose anti-AMPAR encephalitis (7). The main treatment includes first-line therapies, such as corticosteroids, intravenous immunoglobulin, and plasma exchange or immunoadsorption, and second-line regimens, such as mycophenolate, rituximab, and cyclophosphamide. For patients with tumor-associated AE, tumor-targeting treatment should be initially considered (8). A systematic review on anti-AMPAR antibody encephalitis suggested that 84% of patients achieved sustained improvement of symptoms, whereas several other studies reported high rates of neurological relapse (9). The patient in this case report had antibodies targeting AMPA1-R and AMPA2-R in the CSF, with AMPA2-R in the serum. After immunotherapy, the symptoms improved significantly.

AMPARs are heterotetramers composed of GluA1-A4 and mediate most of the fast excitatory synaptic transmission in the brain (2). AMPAR trafficking is a key mechanism that induces nascent synaptic development. The type, threshold, and extent of synaptic plasticity at any synapse are determined by the characteristics and properties of AMPAR (10). GluA1/A2 is primarily located in the limbic system and hippocampus, indicating that GluA1/A2 is closely associated with learning, memory, personality, and epilepsy. A study investigating the effect of AMPAR antibodies on the cultures of live rat hippocampal neurons revealed that the antibodies from patients could lead to a selective decrease in total surface area and synaptic localization of AMPARs, resulting in decreased inhibitory synaptic transmission and increased intrinsic excitability. Furthermore, these changes might be attributed to memory deficits and epilepsy (11). However, interestingly, more than half of the cases have been admitted to the hospital with an initial symptom of memory decline or cognitive impairment (5).

The patient only had rapid memory loss as the first symptom. Because of living separately from their parents, his children were not very aware of the patient's condition in recent years. His wife reported that the patient forgot to buy things occasionally in the last 2 years, but their life was not affected. Thus, AE was the tentative diagnosis; however, there was the possibility of AD or preclinical AD, with some factors leading to an acute exacerbation of the disease process. Testing CSF biomarkers is the main diagnostic tool for detecting AD, as positron emission tomography is expensive (12). Surprisingly, the patient had biomarkers suggestive of AD within the CSF (Aβ_1−42_↓, Aβ_1−42_ /Aβ_1−40_↓, t-Tau↑, and p-Tau↑). According to the AT(N) system proposed by the 2018 National Institute on Aging and Alzheimer's Association workgroup, the results were consistent with the biomarker category of AD (A+: aggregated Aβ, T+: neurofibrillary tangles, N+: neurodegeneration, or neuronal injury) (13).

Accumulating data suggest that AD is a continuous process, and the progression of biomarkers is also a continuum that begins before the onset of symptoms (13). AMPAR and AMPA signaling pathway disorders have been demonstrated to be particularly prominent in the pathogenesis of this continuous process. Changes in actin cytoskeleton integrity and the structure and number of dendritic spines, which occur early in AD, are associated with a decline in AMPA signaling. In addition, AMPA dysfunction correlates with the presence of soluble, but not insoluble, Aβ and Tau species (14). At the same time, anti-AMPAR antibodies can reduce the expression of AMPARs (11). It seems that the pathologic processes of AE and AD might affect each other. One previous study reported that the concentration of progranulin could be a CSF biomarker of NMDAR-AE, and high levels of t-Tau might suggest a risk for hippocampal sclerosis (15). However, the available evidence can only demonstrate that either the presence of antibodies or neurodegeneration might be associated with AE; nevertheless, it is still necessary to show whether anti-AMPAR antibodies could cause a typical change in the CSF biomarkers of AD. Moreover, it should be noted that diabetes and hypertension are associated with neurodegeneration. Insulin resistance and decreased insulin activity can suppress protein kinase B signaling, leading to dephosphorylation and activation activity of glucose synthase kinase-3β, which is involved in Tau phosphorylation and formation of Aβ1–40 and Aβ_1−42_ (16). Cerebral ischemia can stimulate the expression of presenilin, which is involved in Aβ synthesis, leading to the accumulation of Aβ (17). Therefore, further investigations are necessary to determine whether there is a direct link between anti-AMPAR antibodies and alterations in AD pathologic changes or whether the presence of Aβ and p-Tau is a potential predisposing factor for AE.

Besides, it should be noted that patients with AE, who are associated with anti-AMPAR, usually exhibit limbic system involvement or temporomesial abnormality in brain MRI and slow waves or epileptic waves in EEG. Approximately 64% of cases are associated with tumors, while the current case exhibited normal brain MRI. A few cases with short memory loss or confusion as initial symptoms were reported to have normal MRI with normal or general slowing in EEG (3, 5). In this group of patients, especially those with subacute onset or longer duration of disease, the diagnosis seems to be confirmed only by determining autoimmune antibodies. The cognitive impairment in patients with AE or autoimmune dementia is manifested with a rapid disease process, whereas symptoms in patients with AD have a gradual on set over months to years (8). It is generally accepted that the pathogenesis of dementia, and AD, in particular, is associated with autoimmunity, including classic autoantibodies and functional autoantibodies (18). Therefore, we suggest that irrespective of whether anti-AMPAR antibodies are involved in pathologic changes of AD and whether patients with AE exhibit cognitive impairment, they could suffer from AD pathologic changes. However, limited reports are available on anti-AMPAR–associated autoimmune dementia, as well as the relationship between anti-AMPAR antibodies and the changes of CSF. Thanarajah et al. reported an atypical AE case with neuropil antibodies against an unknown epitope, increased Tau level, decreased level of amyloid ratio, and temporoparietal atrophy; however, they did not explore the potential correlation between them (19). Because of a lack of clear-cut history of cognition and typical imaging presentation, whether the case presented here really suffers from AD or pre-clinical AD is uncertain. During 5 months of follow-up, the patient exhibited continuous improvements in cognitive function and was in the stage of mild cognitive impairment. His son confirmed that his father was only more likely to forget things than the period before the onset of the disease. Based on the patient's recovery, we believe that his symptoms of severe cognitive decline were caused by AE and multiple factors that induced amyloid and Tau abnormalities. At the same time, we hypothesize that the patient might have a chronic mild cognitive dysfunction that was exacerbated by the antibodies. Therefore, we have planned to follow up the patient for a long time, including repeated neuropsychological evaluation, analysis of CSF biomarkers half a year later, and brain MRI examinations at least once a year, to observe changes in the patient's condition and any changes toward AD.

In conclusion, we discussed the clinical characteristics and molecular mechanisms of anti-AMPAR encephalitis and its potential interaction with changes in CSF biomarkers of AD, which need to be further investigated. This case provides us with the insight that two pathological processes might coexist. Given the possibility of reversibility and preventing disability, clinicians should be aware of the likelihood of AE when dealing with patients with rapid cognitive decline. However, in these patients and the elderly subjects, in particular, with the possibility of concomitant chronic and progressive cognitive decline, preclinical AD should not be ignored.

## Data Availability Statement

The original contributions presented in the study are included in the article/Supplementary Material, further inquiries can be directed to the corresponding author/s.

## Ethics Statement

Written informed consent was obtained from the individual(s) for the publication of any potentially identifiable images or data included in this article.

## Author Contributions

YS and SC: wrote the manuscript and made table and figures. YS, SC, JG, and WX: reviewed the literature. YS, SC, JG, JL, WX, XL, and JC: performed final manuscript review and editing. All authors contributed to the article and approved the submitted.

## Conflict of Interest

The authors declare that the research was conducted in the absence of any commercial or financial relationships that could be construed as a potential conflict of interest.
