# Supplementary material for: Case Report: Coexistence of Anti-AMPA Receptor Encephalitis and Positive Biomarkers of Alzheimer's Disease
Source: Front Neurol. 2021 Jul 2;12:673347. doi: 10.3389/fneur.2021.673347 (PMC8283122; doi:10.3389/fneur.2021.673347)
Supplement: Supplementary file 1 [file Data_Sheet_1.zip › Data Sheet 1/Details about the laboratory evaluation.pdf]

- Blood routine, urine routine, stool routine, blood biochemistry indexes, coagulation function and screening for infectious diseases:

Abnormal: calcium: 2.09 mmol/L, normal range 2.15-2.57 mmol/L; apolipoprotein B: 0.58 g/L, normal range 0.6-1.1 g/L; apolipoprotein E: 2.1 g/L, normal range 2.9-5.3 g/L; APTT: 36.70 s, normal range 25.1-36.5 s.

Normal: others

- Hemoglobin A1C: 6.30 %, normal range 4.0-6.0%

- Alzheimer-associated neuronal thread protein (AD7c-NTP) in the urine: 1.3ng/ml, normal range: <1.5ng/ml

- Screening for common anti-neuronal and anti-neuropil antibodies:

Positive: CSF: AMPA1-R (titer 1:3.2), AMPA2R (titer 1:320); serum: AMPA2-R (titer 1:100)

Negative: CSF: NMDAR, GABABR, LGI1, CASPR2

Serum: AMPA1-R, NMDAR, GABABR, LGI1, CASPR2, Hu, Yo, Ri (ANNA2), CV2, Amphiphysin, Ma1, Ma2, SOX1, Tr (DNER), Zic4, GAD65, Titin, Recoverin, PKCγ,AQP4, MOG, MBP, GFAP, AQP1, Flotillin-1/2.

- Determinations made in CSF:

Normal: Red blood cell count, nucleated leukocyte count, glucose and chlorine were normal. Pathology showed no significant abnormalities and no cryptococcus or heterotypic cells. CSF PCR for herpes simplex virus 1 and 2.

Abnormal: protein: 0.52 g/L, normal range 0.20-0.40 g/L; immunoglobulin G: 46.1 mg/L, normal range 0-34 mg/L

- Thyroid hormone combination:

Normal: Triiodothyronine, tetraiodothyronine, free triiodothyronine, free tetraiodothyronine, thyroid-stimulating hormone, thyroglobulin antibody, thyroid peroxidase antibody and thyrotropin receptor antibody.

Abnormal: thyroglobulin: 3.37μg/L, normal range: 3.5-77μg/L

- Anticardiolipin antibody:

Normal: Anticardiolipin antibody IgG, anticardiolipin antibody IgM and anticardiolipin antibody IgA

- The spectrum of antinuclear antibodies:

Normal: Anti-nucleosome antibodies, anti-dsDNA antibodies and anti-histone antibodies, anti-SM antibodies, anti-U1nRNP antibodies, anti-SSA60 antibodies, anti-SSA52 antibodies, anti-SSB antibodies, anti-ScL-70 antibodies, anti-alpha-synuclein antibodies, anti-Jo-1 antibodies, anti-ribosomal P protein antibodies and anti-cyclic citrullinated peptide antibodies

- Tumor marker:

Normal: AFP, CEA, SCC, CYFRA21-1, CA19-9, CA72-4, CA24-2, CA50, NSE and PSA

- EEG:

EEG waves are mainly long-range, low to medium amplitude, 6-7Hz θ waves; Extensive moderate abnormality with θ wave as the background.

The images of the EEG were as follows:

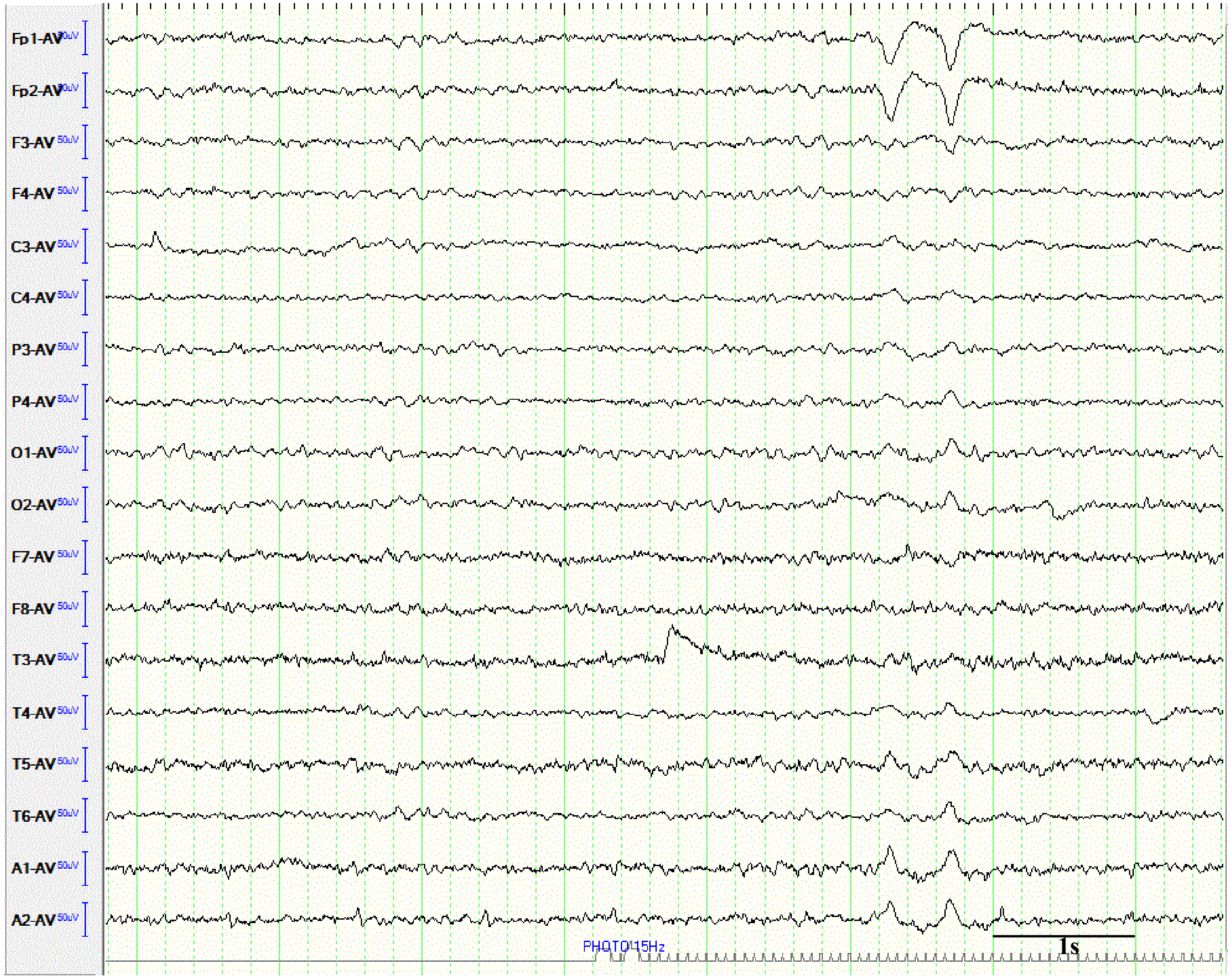
Fp1-AV
Fp2-AV
F3-AV
F4-AV
C3-AV
C4-AV
P3-AV
P4-AV
O1-AV
O2-AV
F7-AV
F8-AV
T3-AV
T4-AV
T5-AV
T6-AV
A1-AV
A2-AV
50uV
PHOTO 15Hz
1s

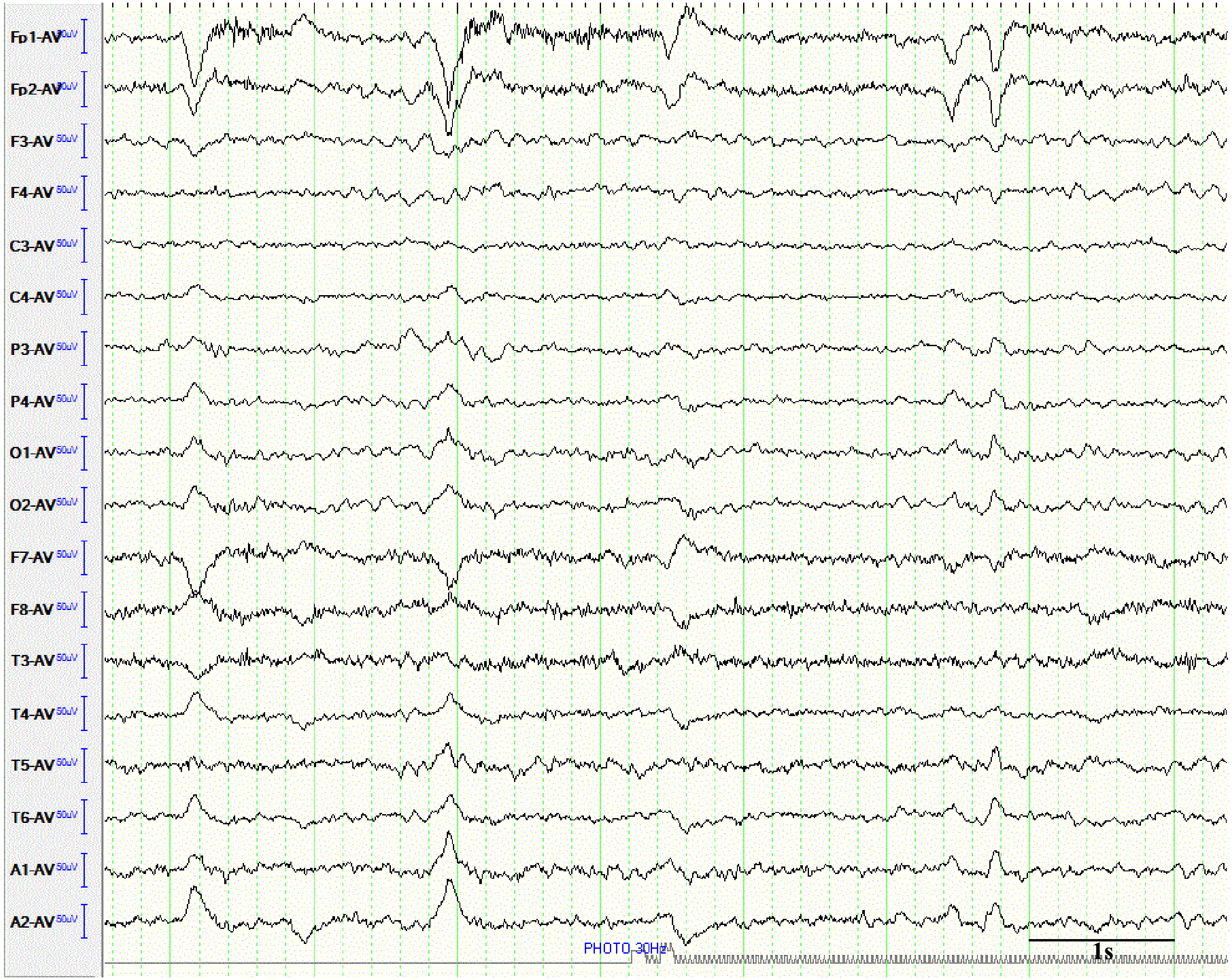

Fp1-AV
Fp2-AV
F3-AV
F4-AV
C3-AV
C4-AV
P3-AV
P4-AV
O1-AV
O2-AV
F7-AV
F8-AV
T3-AV
T4-AV
T5-AV
T6-AV
A1-AV
A2-AV
50uV
PHOTO 30Hz
1s
