# Supplementary material for: Case Report: Coexistence of Anti-AMPA Receptor Encephalitis and Positive Biomarkers of Alzheimer's Disease
Source: Front Neurol. 2021 Jul 2;12:673347. doi: 10.3389/fneur.2021.673347 (PMC8283122; doi:10.3389/fneur.2021.673347)
Supplement: Supplementary file 1 [file Data_Sheet_1.zip › Data Sheet 1/The method of the ELISA determinations.pdf]

CSF was collected in the morning after a minimum 12-hour fast. A Sprotte spinal needle was inserted into the L3-4 or L4-5 vertebral interspace and 22 mL of CSF was removed via gentle extraction into polypropylene syringes. Within 30 minutes of collection, the CSF was combined, gently mixed, centrifuged at 2000g for 10 min to remove red blood cells or other debris, aliquoted into 0.5 mL polypropylene tubes, and stored at −80°C. Samples were analyzed at the Neuropsychiatry Research Institution of the Affiliated Brain Hospital of Nanjing Medical University.

CSF Aβ1-42, Aβ1-40, total tau (t-tau), and phosphorylated tau (P-tau) were analyzed using the Amyloid-beta (1-42) CSF ELISA kit (Catalog-No: RE59661), Amyloid-beta (1-40) Highly Sensitive ELISA kit (Catalog-No: RE59781), hTAU total ELISA kit (Catalog-No:0108000101) and Phospho-TAU ELISA kit (Catalog-No: 30121609) (manufacturer IBL International GmbH, Hamburg, Germany). Measure optical density with a photometer at 450 nm within 15 min after pipetting the Stop Solution. Performance criteria were inter-and intra-assay, and inter-lot imprecision. An Aβ1-42 value < 550 pg/ml or an Aβ1-42/Aβ1-40 ratio ≤0.05 was used to define elevated Aβ, as well as an t-Tau value > 399 pg/ml or an p-Tau valve > 50 pg/ml was used to define elevated Tau protein, based on prior receiver operating characteristic analyses showing these values best discriminated cognitively healthy adults from individuals with dementia and the reference range recommended by the German Society for CSF diagnostics and Clinical Neurochemistry e. V. in the German Society of Neurology[1-4].

All assays employ the sandwich ELISA principle.

The Amyloid-beta (1-42) CSF ELISA kit and the Amyloid-beta (1-40) Highly Sensitive ELISA kit use a monoclonal antibody either directed against the C-terminus of the Aß1-40 peptide or against the C-terminus of the Aß1-42 peptide, which are coated onto the surface area of the microtiter plate. The presence of the captured peptides (Aß1-40 or Aß1-42) is detected by the concomitant binding by the N-terminus specific monoclonal antibody (clone 82E1) conjugated with a horseradish peroxidase (HRP). Tetramethylbenzidine (TMB) is used as a chromogenic substrate.

The hTAU total ELISA kit uses a monoclonal antibody specific for the amino acid region 160-180 of Tau441, immobilized on the surface area of the microtiter plate. Tau protein from samples, standards and controls binds to this antibody and Tau is detected by another specific Tau protein binding monoclonal antibody. The binding of the monoclonal antibody is detected via a conjugated horseradish peroxidase using the chromogenic substrate TMB. The concentration of TAU is proportional to the obtained optical density.

The Phospho-TAU ELISA kit uses a monoclonal antibody specifically binding phosphorylated tau protein, immobilized on the surface area of the microtiter plate. Phosphorylated tau protein from samples, standards and controls binds to this antibody and target is detected by another anti-tau monoclonal anti-body horseradish peroxidase conjugated. Amount of bound conjugated antibody is estimated using chromogenic substrate TMB. The concentration of phosphorylated tau protein is proportional to the obtained optical density.

Reference

1 Alcolea, D. *et al.* Amyloid precursor protein metabolism and inflammation markers in preclinical Alzheimer disease. *Neurology* **85**, 626-633, doi:10.1212/wnl.0000000000001859 (2015).

2 Lewczuk, P., Lelental, N., Spitzer, P., Maler, J. & Kornhuber, J. Amyloid-β 42/40 cerebrospinal fluid concentration ratio in the diagnostics of Alzheimer's disease: validation of two novel assays. *Journal of Alzheimer's disease : JAD* **43**, 183-191, doi:10.3233/jad-

140771 (2015).

3 Mulder, C. *et al.* Amyloid-beta(1-42), total tau, and phosphorylated tau as cerebrospinal fluid biomarkers for the diagnosis of Alzheimer disease. *Clin Chem* **56**, 248-253, doi:10.1373/clinchem.2009.130518 (2010).

4 Zwan, M. D. *et al.* Use of amyloid-PET to determine cutpoints for CSF markers: A multicenter study. *Neurology* **86**, 50-58, doi:10.1212/wnl.0000000000002081 (2016).
